# Placental Size Is Associated with Mental Health in Children and Adolescents

**DOI:** 10.1371/journal.pone.0040534

**Published:** 2012-07-09

**Authors:** Natasha Khalife, Vivette Glover, Anna-Liisa Hartikainen, Anja Taanila, Hanna Ebeling, Marjo-Riitta Järvelin, Alina Rodriguez

**Affiliations:** 1 Department of Epidemiology and Biostatistics, Imperial College London, London, United Kingdom; 2 Institute of Reproductive and Developmental Biology, Imperial College London, London, United Kingdom; 3 Institute of Clinical Medicine, University of Oulu, Oulu, Finland; 4 Institute of Health Sciences, University of Oulu, Oulu, Finland; 5 Unit of General Practice, Oulu University Hospital, Oulu, Finland; 6 Clinic of Child Psychiatry, Oulu University Hospital, Oulu, Finland; 7 Medical Research Council Health Protection Agency Centre for Environment and Health, Imperial College London, London, United Kingdom; 8 National Institute for Health and Welfare, Oulu, Finland; 9 Biocenter Oulu, University of Oulu, Oulu, Finland; 10 Department of Social Sciences – Psychology, Mid Sweden University, Östersund, Sweden; 11 Medical Research Council Social Genetic Developmental Psychiatry Centre, King's College London, London, United Kingdom; UCL Institute of Child Health, University College London, United Kingdom

## Abstract

**Background:**

The role of the placenta in fetal programming has been recognized as a highly significant, yet often neglected area of study. We investigated placental size in relation to psychopathology, in particular attention deficit hyperactivity disorder (ADHD) symptoms, in children at 8 years of age, and later as adolescents at 16 years.

**Methodology/Principal Findings:**

Prospective data were obtained from The Northern Finland Birth Cohort (NFBC) 1986. Placental weight, surface area and birth weight were measured according to standard procedures, within 30 minutes after birth. ADHD symptoms, probable psychiatric disturbance, antisocial disorder and neurotic disorder were assessed at 8 years (n = 8101), and ADHD symptoms were assessed again at 16 years (n = 6607), by teachers and parents respectively. We used logistic regression analyses to investigate the association between placental size and mental health outcomes, and controlled for gestational age, birth weight, socio-demographic factors and medical factors, during gestation. There were significant positive associations between placental size (weight, surface area and placental-to-birth-weight ratio) and mental health problems in boys at 8 and 16 years of age. Increased placental weight was linked with overall probable psychiatric disturbance (at 8y, OR  = 1.14 [95% CI  = 1.04–1.25]), antisocial behavior (at 8 y, OR  = 1.14 [95% CI  = 1.03–1.27]) and ADHD symptoms (inattention-hyperactivity at 16y, OR  = 1.19 [95% CI  = 1.02–1.38]). No significant associations were detected among girls.

**Conclusions/Significance:**

Compensatory placental growth may occur in response to prenatal insults. Such overgrowth may affect fetal development, including brain development, and ultimately contribute to psychopathology.

## Introduction

Mental health problems, in particular attention deficit hyperactivity disorder (ADHD) symptoms, are a significant cause of functional disability in children and adolescents [1], [2]. While genetic and childhood environmental factors have been studied extensively in relation to the development of psychiatric disorders, accumulating evidence has revealed that prenatal factors are potentially another powerful source of influence [3], in accordance with fetal programming [4], [5]. Indeed, an adverse intrauterine environment has been associated with a range of mental health problems in children, including ADHD symptoms, anxiety and antisocial disorder [6]. However, our understanding of the mechanisms linking prenatal exposures to later health outcomes is very limited. It has recently been suggested that the placenta may be a significant component in translating maternal influences during pregnancy, consequently affecting fetal development and thereby adult health [7], [8]. The placenta, providing an interface between the mother and fetus, appears to be in a key position to mediate fetal programming. Evidence from recent studies indicates that the placenta responds to disturbances in the maternal environment with a range of structural and functional adaptations, including changes in placental growth [8], [9]. Abnormal placental growth is associated with altered fetal nutrient and hormone supply [5], which in turn may induce adaptations in the fetus, thereby programming an increased risk of developing disease in adult life [8]. In light of this, it is surprising that the role of the placenta in the programming of psychopathology has received limited research attention. To our knowledge, only one previous study has examined the relation between placental size and mental health, which found that small placental weight was associated with schizotypal traits in women at 31 years of age [10]. To date, it is not known whether placental size is related to mental health in children and adolescents.

Here we analysed prospective data from a large, longitudinal cohort to investigate whether characteristics related to placental size – placental weight, surface area and placental-to-birth-weight ratio, are associated with mental health, in particular ADHD symptoms, in childhood and adolescence. We investigated sexual dimorphism, due to well-established sex differences in behavior and the placenta. Besides the higher prevalence of behavioral problems among boys compared to girls [11], male placentas are more vulnerable to maternal undernutrition, and more readily undergo compensatory placental growth in response to such insults [12]. We hypothesise that placental size will be related to psychopathology, in particular ADHD symptoms, during childhood (8 years) and later in adolescence (16 years), after controlling for relevant confounders.

## Methods

### Ethics Statement

The ethics committee of Northern Ostrobotnia Hospital District approved the study, and both parents and adolescents gave written informed consent.

### Cohort

**Figure 1:**
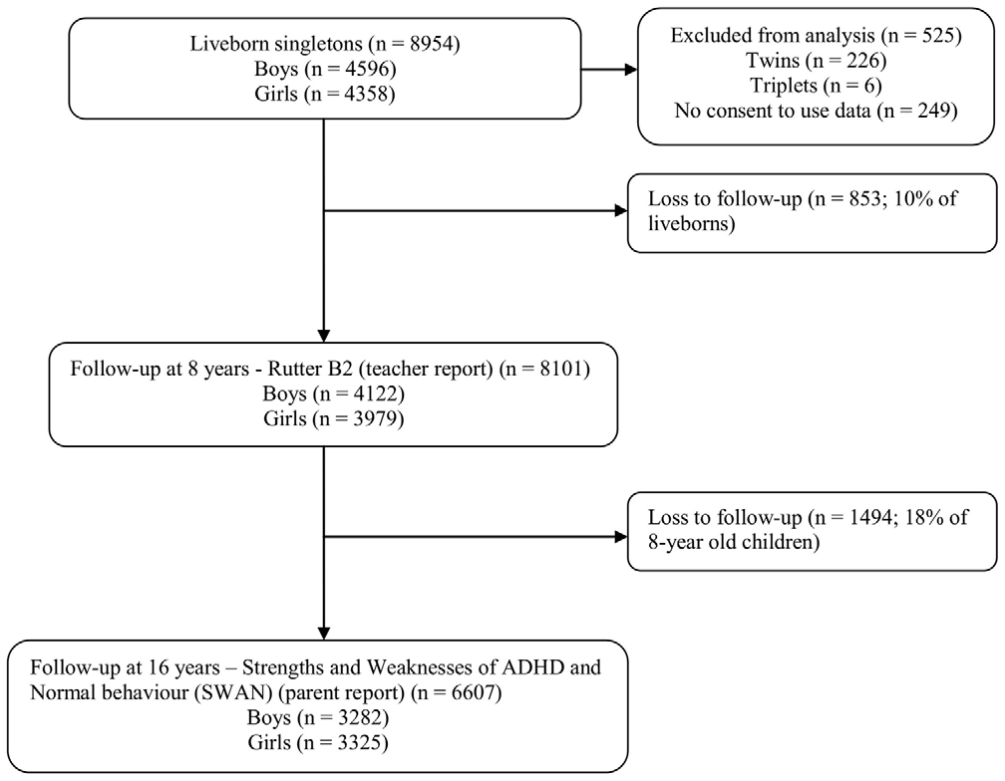
Flowchart of study participants.

Data were obtained from The Northern Finland Birth Cohort (NFBC) 1986, which consists of 9479 children born in Oulu and Lapland provinces, who were studied prospectively from early pregnancy until 16 years of age (Figure 1). Children with an expected date of birth between July 1, 1985 and June 30, 1986 were eligible; 99% of eligible births in the study area were included.

Pregnant women were recruited at maternity health centres, at their first antenatal visit (approximately gestational week 12), and provided background information using structured self-report questionnaires, which were returned by gestational week 24, if still pregnant. Antenatal clinical and birth outcome data were obtained from maternity health centres and hospital medical records (completed by midwives during pregnancy and at birth), and abstracted onto study forms.

After excluding multiple birth children (226 twins and 6 triplets) and those who had not consented use of their data (n = 249), data from 8954 liveborns (4596 boys) were available for analysis (Figure 1). When children were 8 years of age, teachers were asked to complete questionnaires regarding child behavior. Of children still residing in Finland, 90% (n = 8101) of the teachers completed the questionnaire. At 16 years of age, 74% (n = 6607) of families still residing in Finland provided information on adolescent behavior.

### Variables

#### Placental Characteristics

Within 30 minutes following delivery, placentas were washed with water, cleaned from blood clots, and then weighed to the nearest gram. Placental weight included membranes and the umbilical cord, cut approximately 5 cm from the neonate. Whilst spread out on a plane, placental breadth and length were used to measure placental surface area (maternal side) in centimetres squared. The placental-to-birth-weight ratio was calculated by dividing placental weight by birth weight, and then multiplying by 100 to produce a percentage of placental weight relative to birth weight.

#### Child and Adolescent Behavior

Teachers assessed child behavior at the age of 8 years using the Rutter B2 scale [13], a well-validated screener for childhood psychopathology. Each of the 26 items is rated as either it ‘certainly applies’ (scored  = 2), ‘applies somewhat’ (scored  = 1) or ‘does not apply’ (scored  = 0); yielding a total score between 0 to 52. According to the screening criteria, a total score of ≥9 indicates probable psychiatric disturbance in general. The questionnaire generates three sub-scores: the neurotic sub-score (the sum of four items – often worried, miserable, fearful and tears on arrival at school), the antisocial sub-score (the sum of six items – destructive, fights, disobedient, lies, steals and bullies) and the inattention-hyperactivity sub-score (the sum of three items – restless, squirmy and fidgety, and poor concentration ). Probable positive screen for neurotic disorder is identified by a total score of ≥9 and a higher score on neurotic items versus antisocial items, whereas antisocial disorder is identified by a total score of ≥9 and a higher score on antisocial items versus neurotic items. Probable combined inattention-hyperactivity disorder is defined as a total score of ≥9 and sum of all three inattention-hyperactivity items ≥3. Additionally, we examined the core ADHD symptoms individually i.e. inattention (item ‘poor concentration’ ≥1) and hyperactivity (sum of items ‘restless’ and ‘squirmy and fidgety’ ≥3).

At 16 years of age, parents reported adolescents' behavior using the Strengths and Weaknesses of ADHD symptoms and Normal behavior (SWAN) scale [14]. The SWAN is an 18-item scale, based on the symptoms of ADHD listed in the DSM-IV. As this scale measures both weaknesses (scored as 3, 2 and 1) and strengths (scored as −1, −2 and −3), along with average behavior (scored as 0), it is expected to produce a normal distribution of behavioral scores, thereby reducing the risk of over-identifying youths as screening positive for ADHD. The inattentive subscale and hyperactive-impulsivity subscale each comprise 9 items, and the combined subscale contains all items. The 95^th^ percentile of the distribution of mean scores on each subscale was used as a cut-off point to identify adolescents with ADHD symptoms as probable clinical cases. The cut-off values for the inattention, hyperactive-impulsivity and combined subscales were respectively 0.625, 0.125 and 0.277, previously described in detail [15].

#### Confounders

We considered potential confounders which may be related to placental size and mental health outcomes, as indicated by our descriptive analysis as well as the literature. These were gestational age (weeks) [16], birth weight (grams) [17] as well as maternal socio-demographic and medical factors [16], [18], [19], [20], all of which were collected prospectively. The socio-demographic factors were maternal age (years), education (either <11 years of education or ≥11 years of education, coded as 0 or 1, respectively), family structure (either married/co-habiting or single/widowed/divorced, coded as 0 or 1, respectively) and social class (by occupation; either class 1 (professional, upper/lower white collar or farmer ≥8 hectares of land) or class 2 (unskilled worker or farmer <8 hectares of land), coded as 0 or 1, respectively). This dichotomized social class variable was based on paternal occupation (or maternal occupation, if missing data or single mother), which was transformed to an indicator of social class according to a national system of classifications and standards in Finland [21]. The medical factors controlled for were parity (included as a continuous variable), pre-pregnancy body mass index (BMI) (pre-pregnancy weight [kg]/height^2^ [m^2^]) (included as a continuous variable), gestational weight gain (weight at last antenatal appointment [kg] – pre-pregnancy weight [kg]), and smoking during pregnancy (either no or yes, coded as 0 and 1, respectively).

### Statistical Analyses

We initially performed correlation and ANOVA analyses to identify any relations between placental size and potential confounders. Logistic regression analyses were used to investigate the association between placental size (placental weight, surface area and placental-to-birth-weight ratio) and mental health outcomes in children and adolescents. The predictors – the placental characteristics, were included as continuous variables. Placental weight and surface area were initially analysed continuously in 1 g and 1 cm^2^ increments, respectively. To facilitate clinical interpretation of the results, the main analysis included placental weight and surface area as continuous variables in 100 g and 10 cm^2^ increments, respectively. All the mental health outcomes were dichotomized, coded as 1 to indicate the presence of mental health problems (and 0 if absent), according to the criteria of each screening instrument. We controlled for gestational age, birth weight, socio-demographic factors (maternal age, education, family structure and social class), and medical factors (parity, pre-pregnancy BMI, gestational weight gain and smoking during pregnancy).

To investigate non-linear associations, we repeated the analysis with stratified placental data (8 groups: <400 g, 400–499 g, 500–599, 600–699 g, 700–799 g, 800–899 g, 900–999 g and ≥1000 g). Furthermore, to assess frequency distributions for descriptive purposes, placental weight was stratified according to its distribution in the study population, such that three categories were formed: <550 g (represents <25^th^ percentile), 550–719 g (represents 25^th^–75^th^ percentiles) and >720 g (represents >75^th^ percentile).

Using SPSS 17.0, all analyses were conducted separately for males and females, due to sex differences in placentas and behavioral traits.

## Results

Table 1 shows birth and child/adolescent behavioural outcomes. For the three categories of placental weight, <550 g, 550–719 g and >720 g, the weight ranges from the mean were 422–530 g, 578–676 g and 726–902 g, respectively, for the entire study sample.

**Table 1:** Birth and child/adolescent characteristics presented as means ± SD or n (%).

Characteristic	Mean ± SD or n (%)			n
	All	Male	Female	
**Birth outcomes**				
Sex		4596 (51.3)	4358 (48.7)	8954
Birth weight (g)	3575±534	3638±537	3509±523	8954
<2500	2044±449	2025±459	2059±441	248
2500–4499	3578±424	3626±420	3528±422	8388
≥4500	4704±194	4707±197	4697±190	318
Gestational age at birth (weeks)	39.4±1.6	39.4±1.6	39.5±1.6	8950
Preterm birth (<37 weeks)	34.4±2.3	34.4±2.3	34.3±2.3	366
Term birth (≥37 weeks)	39.6±1.2	40.0±1.2	40.0±1.2	8584
Placental weight (g)	646±132	652±133	640±131	8934
<550		874 (48.6)	924 (51.4)	1798
550–719		2425 (50.8)	2349 (49.2)	4774
≥720		1287 (54.5)	1075 (45.5)	2362
Placental surface area (cm^2^)	335±70	337±70	334±69	8821
Placental-to-birth-weight ratio	18.2+3.1	18.0±3.1	18.3±3.0	8934
**Behavioral outcomes**				
***8-y-olds (teacher report)***				
**Rutter** a				
Probable psychiatric disturbance	1140 (14.1)	801 (19.5)	339 (8.6)	8065
Antisocial disorder	725 (9.0)	570 (13.9)	155 (4.0)	8065
Neurotic disorder	328 (4.1)	171 (4.2)	157 (4.0)	8065
Inattention-hyperactivity	756 (9.4)	576 (14.0)	180 (4.5)	8080
Inattention^b^	1705 (21.1)	1220 (30.0)	485 (12.2)	8088
Hyperactivity^c^	575 (7.1)	468 (11.4)	107 (2.7)	8086
***16-y-olds (parent report)***				
**SWAN** a				
Combined ADHD	349 (5.3)	231 (7.0)	118 (3.5)	6607
Inattention^d^	332 (5.1)	230 (7.1)	102 (3.1)	6500
Hyperactivity-impulsivity^e^	306 (4.7)	197 (6.1)	109 (3.3)	6467

a assessment of symptoms based on fulfilment of criteria according to the Rutter B2/Strengths and Weaknesses of ADHD symptoms and Normal behavior (SWAN) scale.

b assessment based on Rutter item number 16.

c assessment based on sum of Rutter items 1 and 3.

d SWAN subscale consisting of sum of 9 items.

e SWAN subscale consisting of sum of 9 items.

Correlation analyses showed that placental size was significantly associated with the potential confounders. Placental weight significantly correlated with gestational age (r = .23, p<.01), birth weight (r = .65, p<.01), maternal socio-demographic factors (except for education): age (r = .08, p<.01), family structure at birth (r = −.05, p<.01), social class by occupation (r = .02, p<.05), and medical factors: parity (r = .11, p<.01), pre-pregnancy BMI (r = .18, p<.01), gestational weight gain (r = .14, p<.01) and smoking during pregnancy (r = −.05, p<.01). Table 2 shows significant associations between mean placental weight and each of the potential confounders (except for maternal education). Mean placental weight differed significantly between the dichotomized mental health outcomes specifying antisocial disorder (males and entire sample) and neurotic disorder (females) at 8 years, and inattention symptoms (entire sample) at 16 years.

**Table 2:** Mean placental weight according to potential confounders and child/adolescent mental health outcomes.

Potential Confounders and Mental Health Outcomes	Mean Placental Weight (g) (SD)					
	n (%)	All	n (%)	Male	n (%)	Female
**Potential Confounders**						
Birth weight (g)	8934		4586		4348	
<2500	247 (2.8)	448 (106)	110 (2.4)	455 (108)	137 (3.2)	443 (105)
2500–4499	8370 (93.7)	644 (121)	4259 (92.9)	646 (121)	4111 (94.5)	641 (121)
≥4500	317 (3.5)	860 (138)	217 (4.7)	837 (139)	100 (2.3)	869 (136)
p^a^		.000		.000		.000
Gestational age at birth (weeks)	8930		4582		4348	
<37	363 (4.1)	524 (163)	189 (4.1)	555 (171)	174 (4.0)	528 (154)
37–41	8226 (92.1)	650 (129)	4213 (92)	655 (130)	4013 (92.3)	644 (127)
≥42	341 (3.8)	600 (127)	180 (3.9)	663 (124)	161 (3.7)	656 (131)
p^a^		.000		.000		.000
Maternal age (years)	8934		4586		4348	
<20	655 (7.3)	636 (125)	336 (7.3)	645 (120)	319 (7.3)	626 (129)
20–34	7105 (79.5)	645 (131)	3635 (79.3)	651 (133)	3470 (79.8)	639 (129)
≥35	1174 (13.1)	654 (142)	615 (13.4)	657 (143)	559 (12.9)	652 (141)
p^a^		.012		.381		.019
Maternal education (years)	7855		3999		3856	
<11	2377 (30.3)	651 (138)	1184 (29.6)	657 (136)	1193 (30.9)	645 (139)
≥11	5478 (69.7)	645 (127)	2815 (70.4)	651 (129)	2663 (69.1)	640 (125)
p^a^		.089		.172		.265
Family structure	8908		4570		4338	
Married/cohabiting	8453 (94.9)	647 (132)	4347 (95.1)	654 (133)	4106 (94.7)	641 (131)
Single/widowed/divorced	455 (5.1)	620 (127)	223 (4.9)	614 (123)	232 (5.3)	625 (130)
p^a^		.000		.000		.069
Family social class (by occupation)	8646		4438		4208	
I						
Professional	521 (6.0)	647 (132)	265 (6.0)	647 (142)	256 (6.1)	647 (121)
Upper white collar	1723 (20.0)	648 (127)	853 (19.2)	659 (131)	870 (20.7)	638 (122)
Lower white collar	3476 (40.2)	644 (131)	1802 (40.6)	651 (130)	1674 (39.8)	638 (131)
Farmer ≥8 hectares	348 (4.0)	666 (146)	178 (4.0)	676 (154)	170 (4.0)	655 (136)
II						
Unskilled worker	2525 (29.2)	642 (133)	1319 (29.7)	645 (131)	1206 (28.7)	640 (135)
Farmer <8 hectares	53 (.6)	672 (178)	21 (.5)	731 (202)	32 (.7)	633 (151)
p^a^		.025		.001		.565
Parity	8895		4565		4330	
0	3029 (34.1)	623 (128)	1551 (34)	629 (128)	1478 (34.1)	617 (128)
1	2969 (33.4)	653 (132)	1485 (32.5)	658 (134)	1484 (34.3)	647 (130)
2	1604 (18.0)	660 (134)	863 (18.9)	669 (136)	741 (17.1)	650 (126)
≥3	1293 (14.5)	666 (132)	666 (14.6)	666 (133)	627 (14.5)	666 (136)
p^a^		.000		.000		.000
Pre-pregnancy BMI (kg/m^2^)	8708		4478		4230	
<20	2136 (24.5)	612 (121)	1138 (25.4)	618 (120)	998 (23.6)	606 (123)
20–24.99	5078 (58.3)	649 (130)	2576 (57.5)	655 (132)	2502 (59.1)	643 (127)
≥25	1494 (17.2)	683 (142)	764 (17.1)	688 (142)	730 (17.3)	677 (141)
p^a^		.000		.000		.000
Gestational weight gain (kg)	8262		4244		4018	
<11	1911 (23.1)	620 (132)	909 (21.4)	623 (137)	1002 (25.0)	617 (128)
11–16.99	4310 (52.2)	645 (126)	2223 (52.4)	650 (125)	2087 (51.9)	640 (126)
≥17	2041 (24.7)	673 (136)	1112 (26.2)	679 (136)	929 (23.1)	666 (130)
p^a^		.000		.000		.000
Smoking during pregnancy	8696		4455		4241	
No	6989 (80.4)	649 (131)	3564 (80.0)	654 (133)	3425 (80.8)	644 (130)
Yes	1707 (19.6)	633 (130)	891 (20.0)	638 (131)	816 (19.2)	627 (129)
p^a^		.000		.001		.001
Number of cigarettes (per day)	939		500		439	
<10 cigarettes	556 (59.2)	630 (121)	293 (58.6)	634 (116)	263 (59.9)	626 (127)
≥10 cigarettes	383 (40.8)	631 (138)	207 (41.4)	641 (138)	176 (40.1)	618 (137)
p^a^		.968		.539		.544
**Mental Health Outcomes**						
**8-y-olds Rutter (teacher report)** b						
Probable psychiatric disturbance	8046		4097		3949	
Yes	1139 (14.2)	651 (138)	800 (19.5)	660 (141)	339 (8.6)	632 (130)
No	6907 (85.8)	646 (129)	3297 (80.5)	651 (127)	3610 (91.4)	642 (129)
p^a^		.245		.089		.145
Antisocial disorder	8046		4097		3949	
Yes	724 (9.0)	659 (142)	569 (13.9)	663 (144)	155 (3.9)	646 (135)
No	7322 (91.0)	646 (129)	3528 (86.1)	651 (128)	3794 (96.1)	641 (129)
p^a^		.010		.046		.683
Neurotic disorder	8046		4097		3949	
Yes	328 (4.1)	641 (133)	171 (4.2)	660 (137)	157 (4.0)	621 (126)
No	7718 (95.9)	647 (130)	3926 (95.8)	652 (130)	3792 (96.0)	642 (130)
p^a^		.419		.417		.041
Inattention- hyperactivity	8061		4103		3958	
Yes	755 (9.4)	650 (138)	575 (14.0)	656 (136)	180 (4.5)	628 (140)
No	7306 (90.6)	647 (129)	3528 (86.0)	652 (129)	3778 (95.5)	642 (129)
p^a^		.568		.442		.163
Inattention^c^	8069		4108		3961	
Yes	1703 (21.1)	650 (135)	1218 (29.6)	655 (135)	485 (12.2)	639 (132)
No	6366 (78.9)	646 (129)	2890 (70.4)	652 (128)	3476 (87.8)	642 (129)
p^a^		.281		.496		.625
Hyperactivity^d^	8067		4105		3962	
Yes	573 (7.1)	654 (137)	466 (11.4)	655 (138)	107 (2.7)	653 (132)
No	7494 (92.9)	646 (129)	3639 (88.6)	652 (129)	3855 (97.3)	641 (130)
p^a^		.159		.677		.354
**16-y-olds SWAN (parent report)** b						
Combined ADHD	6594		3275		3319	
Yes	349 (5.3)	659 (138)	231 (7.1)	668 (144)	118 (3.6)	641 (123)
No	6245 (94.7)	647 (129)	3044 (92.9)	652 (128)	3201 (96.4)	642 (129)
p^a^		.098		.076		.930
Inattention^e^	6488		3227		3261	
Yes	332 (5.1)	663 (138)	230 (7.1)	666 (140)	102 (3.1)	656 (132)
No	6156 (94.9)	646 (128)	2997 (92.9)	652 (127)	3159 (96.9)	641 (129)
p^a^		.024		.110		.256
Hyperactivity-impulsivity^f^	6455		3206		3249	
Yes	306 (4.7)	650 (135)	197 (6.1)	660 (135)	109 (3.4)	632 (133)
No	6149 (95.3)	647 (129)	3009 (93.9)	652 (129)	3140 (96.6)	642 (128)
p^a^		.677		.402		.427

a for heterogeneity, analysis of variance.

b assessment of symptoms based on fulfilment of criteria according to the Rutter B2/Strengths and Weaknesses of ADHD symptoms and Normal behavior (SWAN) scale.

c assessment based on Rutter item number 16.

d assessment based on sum of Rutter items 1 and 3.

e SWAN subscale consisting of sum of 9 items.

f SWAN subscale consisting of sum of 9 items.

Both unadjusted and adjusted logistic regression analyses revealed positive associations between placental size (placental weight, surface area and placental-to-birth-weight ratio – as continuous variables) and mental health outcomes in boys at 8 and 16 years of age, as shown in Table 3. The adjusted analyses largely show stronger and more significant associations between male placental size and mental health outcomes, including overall probable psychiatric disturbance, ADHD symptoms and antisocial disorder in 8-year olds, and ADHD symptoms in 16-year olds. For example, for every 100 g increase in placental weight, the risk for probable psychiatric disturbance at 8 years and combined inattention-hyperactivity at 16 years increased by 14% and 19%, respectively. Furthermore, the adjusted results indicate that compared to placental surface area (10 cm^2^) and placental-to-birth-weight ratio, increased placental weight (100 g) was the strongest predictor of mental health problems in boys at both 8 and 16 years of age. Linear analyses indicated that a respective increase of 1 g and 1 cm^2^ in placental weight and surface area were significantly associated with mental health problems in boys. A 1 g increase in placental weight was associated with probable psychiatric disturbance at 8 years (OR  = 1.001 [95% CI  = 1.000–1.002]) and combined inattention-hyperactivity at 16 years (OR  = 1.002 [95% CI  = 1.000–1.003]), and a 1 cm^2^ increase in placental surface area was associated with inattention-hyperactivity symptoms at 8 years (OR = 1.002 [95% CI = 1.001–1.004]) and inattention symptoms at 16 years (OR = 1.003 [95% CI = 1.000–1.005]). A 10 g increase in placental weight i.e. within 1 SD from the mean, was positively associated with mental health problems in boys (for probable psychiatric disturbance at 8 years, OR  = 1.013 [95% CI  = 1.004–1.023]). In girls, no significant associations were detected between placental size and mental health outcomes at either 8 years or 16 years of age, as shown in Table 4.

**Table 3:** Logistic regression results for the association between male placental size (weight, surface area and placental-to-birth-weight ratio) and mental health outcomes.

Behavior	Male Placental Weight (100g)					Male Placental Surface Area (10cm^2^)					Male Placental-to-Birth-Weight Ratio				
	Unadjusted		Adjusted^a^			Unadjusted		Adjusted^a^			Unadjusted		Adjusted^b^		
	OR	95% CI	n	OR	95% CI	OR	95% CI	n	OR	95% CI	OR	95% CI	n	OR	95% CI
**8-y-olds Rutter (teacher report)**															
Probable psychiatric disturbance	1.05	.99–1.12	3276	1.14**	1.04–1.25	1.01	1.00–1.02	3236	1.01	1.00–1.03	1.04**	1.01–1.07	3276	1.04*	1.01–1.08
Antisocial disorder	1.07*	1.00–1.15	3276	1.14*	1.03–1.27	1.01	1.00–1.02	3236	1.02*	1.00–1.04	1.05**	1.02–1.08	3276	1.04*	1.01–1.08
Neurotic disorder	1.05	.93–1.18	3276	1.19	.99–1.42	1.00	.98–1.02	3236	1.00	.97–1.03	1.04	.98–1.09	3276	1.06	.99–1.13
Inattention-hyperactivity	1.03	.96–1.10	3282	1.11*	1.00–1.24	1.01	1.00–1.02	3242	1.02**	1.01–1.04	1.03	1.00–1.07	3282	1.03	.99–1.07
Inattention	1.02	.97–1.07	3286	1.11*	1.02–1.20	1.00	.99–1.01	3246	1.01	1.00–1.03	1.03**	1.01–1.06	3286	1.03*	1.00–1.06
Hyperactivity	1.02	.94–1.09	3283	1.12*	1.00–1.26	1.01	1.00–1.02	3243	1.03**	1.01–1.05	1.03	.99–1.06	3283	1.04	.99–1.08
**16-y-olds SWAN (parent report)**															
Combined ADHD	1.10	.99–1.21	2754	1.19*	1.02–1.38	1.01	.99–1.03	2724	1.02	1.00–1.05	1.06*	1.01–1.11	2754	1.06*	1.00–1.11
Inattention	1.09	.98–1.20	2720	1.17*	1.00–1.37	1.01	.99–1.03	2691	1.03*	1.01–1.06	1.06*	1.01–1.11	2720	1.05	1.00–1.11
Hyperactivity-impulsivity	1.05	.94–1.17	2701	1.11	.94–1.31	1.01	.99–1.03	2672	1.02	1.00–1.05	1.03	1.00–1.08	2701	1.03	.97–1.09

a adjusted for gestational age, birth weight, socio-demographic factors (maternal age, family structure, education and social class) and medical factors (smoking during pregnancy, parity, pre-pregnancy BMI and gestational weight gain).

b adjusted as above, except for birth weight.

* p<.05; **p<.01.

**Table 4:** Logistic regression results for the association between female placental size (weight, surface area and placental-to-birth-weight ratio) and mental health outcomes.

Behavior	Female Placental Weight (100g)					Female Placental Surface Area (10cm^2^)					Female Placental-to-Birth-Weight Ratio				
	Unadjusted		Adjusted^a^			Unadjusted		Adjusted^a^			Unadjusted		Adjusted^b^		
	OR	95% CI	n	OR	95% CI	OR	95% CI	n	OR	95% CI	OR	95% CI	n	OR	95% CI
**8-y-olds Rutter (teacher report)**															
Probable psychiatric disturbance	.94	.86–1.02	3176	.91	.79–1.04	1.00	.98–1.01	3140	1.00	.97–1.02	.99	.95–1.03	3176	.97	.92–1.01
Antisocial disorder	1.03	.91–1.16	3176	.97	.80–1.20	1.01	.99–1.03	3140	1.01	.99–1.04	1.00	.95–1.06	3176	.98	.92–1.05
Neurotic disorder	.88	.77–.99	3176	.88	.72–1.10	.99	.97–1.02	3140	1.00	.96–1.02	.97	.92–1.03	3176	.96	.89–1.02
Inattention-hyperactivity	.92	.82–1.04	3185	.90	.75–1.1	.99	.97–1.01	3149	.99	.96–1.02	.98	.93–1.03	3185	.95	.90–1.02
Inattention	.98	.91–1.10	3187	1.01	.90–1.13	1.00	.99–1.01	3151	1.01	.99–1.03	1.02	.99–1.10	3187	1.00	.96–1.04
Hyperactivity	1.07	.93–1.24	3189	.95	.76–1.19	1.00	.97–1.03	3153	.99	.95–1.03	.99	.93–1.06	3189	.98	.90–1.06
**16-y-olds SWAN (parent report)**															
Combined ADHD	.99	.86–1.15	2779	.92	.74–1.15	1.01	.98–1.04	2745	1.01	.98–1.05	.99	.93–1.05	2779	.97	.90–1.05
Inattention	1.09	.94–1.27	2737	1.20	.95–1.50	1.01	.98–1.04	2702	1.02	.98–1.06	1.05	.98–1.11	2737	1.06	.98–1.14
Hyperactivity-impulsivity	.94	.81–1.10	2725	.91	.73–1.15	1.00	.97–1.03	2690	1.01	.98–1.05	.98	.92–1.05	2725	.96	.89–1.04

a adjusted for gestational age, birth weight, socio-demographic factors (maternal age, family structure, education and social class) and medical factors (smoking during pregnancy, parity, pre-pregnancy BMI and gestational weight gain).

b adjusted as above, except for birth weight.

After repeating the analysis using stratified placental data, we did not find evidence for a U-shaped association, as small placental size was unrelated to the mental health outcomes.

## Discussion

This is the first study to investigate the relation between placental size and mental health outcomes in children and adolescents. We identified a positive association between placental size (placental weight, surface area and placental-to-birth-weight ratio) and mental health problems in boys; increased placental size was linked with overall probable psychiatric disturbance, ADHD symptoms and antisocial disorder at 8 years of age, and ADHD symptoms at 16 years, after adjusting for known confounders. It is important to note that the magnitude of the associations were small; however, because psychiatric disorders are highly complex – in terms of both etiology and diagnosis, it is expected that any single variable will contribute a small portion of the variance. Our results suggest that variation in placental size may play a role in the causal pathway leading from prenatal exposures to later mental health problems, but further work is required to determine causality.

Placental overgrowth may occur as a compensatory mechanism in response to various maternal prenatal insults [9], [22], [23], [24]. It has been theorized that an enlarged placenta may reduce its supply of nutrients to the fetus [5]; consequent fetal adaptations may lead to permanent structural and physiological changes to developing organs, programming an increased risk of disease. Our results are in line with this, such that we may speculate that increased placental size – a possible consequence of an adverse maternal environment, may alter fetal nutrient supply, which in turn may affect normal brain development [25], [26], increasing the risk of psychiatric problems later in life. In support of this potential mechanism, prenatal psychosocial stress, a common environmental insult, has been associated with increased placental weight [9], and has been directly and independently linked with atypical cerebral laterality [27], [28], and an increased risk of psychiatric problems in children [29], [30]. Furthermore, atypical cerebral lateralization, which has been linked to child and adolescent mental health problems (including ADHD [31], [32]), was detected in 8–9 year old children who had a moderately low birth weight and disproportionately large placenta [33]. On the surface, it may seem that the suggested mechanism implies that an increased placental-to-birth weight ratio would be the strongest predictor of psychiatric outcomes. However, whilst possibly reducing nutrient supply to the fetus, compensatory mechanisms in the expanding placenta may counteract any intrauterine deficiencies, so that a normal birth weight is still achieved despite suboptimal conditions [7]. This concept could explain why we found increased placental weight was the strongest predictor of psychopathology, compared to the other placental characteristics which we studied. Thus, an enlarged placenta may represent an important link between disturbances in the maternal environment and perturbed fetal brain development, with ensuing mental health problems in children and adolescents.

Variation in the size of the placenta affects aspects of its function, in particular the ability to transfer nutrients to the fetus via changes in the exchange surface area [34]; in general, small placentas are associated with small fetuses [35]. Placental size is affected by maternal factors, such as BMI, gestational weight gain and smoking [16], as well as various other medical and socio-demographic factors, as demonstrated by our own results, indicating that the placenta is receptive to the maternal environment, and undergoes changes in size in an effort to maintain fetal development under suboptimal conditions [7]. For example, in response to maternal undernutrition, the placenta may undergo compensatory enlargement [22]; although this adaptation may improve the overall nutrient supply to the fetus, ensuring a normal birth weight is achieved, the relative contribution of specific nutrients to fetal organs may be altered, resulting in the programming of developing organs [7]. Thus, placental compensatory mechanisms may often ensure a normal birth weight is achieved in adverse circumstances, whilst the placenta itself may be markedly affected [36] – reflecting the physiological stresses which occurred during development. Therefore, compared to birth weight and other common indices used to identify suboptimal intrauterine conditions, placental phenotype can provide additional insight into the intrauterine environment and improve our understanding of the processes underlying fetal programming. Furthermore, placental size may enhance the ability to predict later disease, as several lines of evidence suggest [36], [37], [38].

It is interesting to note that placental size was related to a range of mental health problems (including general probable psychiatric disturbance) in this study. This may correspond to the understanding that placental size is sensitive to various maternal influences, and thus possibly represents an archive of gestational insults, which could affect the developing brain non-specifically.

While there is evidence for a U-shaped relation between placental size and various health outcomes [39], here we report a positive linear association, which may be explained by factors related to the direction of placental-fetal growth disproportion, including maternal nutritional status and the timing of prenatal insults. A study by Barker et al. found that in the offspring of tall, middle-class mothers, who were likely to be well nourished, hypertension was related to large placental size [40]. As Finland is a high income country, it is likely that the general nutritional standard of the nation is good. Furthermore, Finland has an exemplary antenatal care system, along with very low infant and maternal mortality rates [41]. Thus, it is likely that women in the NFBC 1986 experienced compensatory placental growth in response to adverse prenatal conditions. Extreme maternal undernutrition at early and late stages of gestation have respectively been associated with increased and reduced placental size [22]. We speculate that prenatal insults related to socio-demographic factors, which tend to be chronic, may induce a trajectory for increased placental size from the start of pregnancy. Acute gestational insults in late pregnancy may alone reduce placental size; however such insults are less common than perhaps chronic stressors, and so the effect of late gestational insults on placental size may be diminished, thereby masking a potential relationship between small placental size and mental health problems.

In the present study, large placental size was associated with psychiatric problems only in boys. Male placentas may be more sensitive to prenatal insults, and more readily undergo compensatory growth in response to such disturbances [12]. Compared to girls, boys grow faster throughout gestation [42], are usually longer at any placental weight and have a smaller placental-to-birth-weight ratio [43]; it has thus been postulated that male placentas are more efficient but have less reserve capacity, causing them to be more vulnerable to undernutrition [12], and presumably to other forms of physiological stress. One study found that in response to maternal asthma during pregnancy, there was no change in the activity of the male placental hydroxysteroid (11-beta) dehydrogenase 2 (HSD11B2) enzyme – the fetoplacental barrier to maternal cortisol, and the fetus continued to grow [44]. In contrast, females showed reduced placental HSD11B2 activity and decreased fetal growth. The authors suggested that the lack of response by the male placenta may contribute to the increased risk of morbidity and mortality of the male fetus. Furthermore, a study in mice demonstrated that male placentas were more vulnerable to prenatal stress, exhibiting an increase in the expression of placental genes related to growth factors [45], which may lead to increased male placental size.

The present study has a number of strengths, including use of prospective data, derived from a large, longitudinal, population-based cohort. Placental measurements were performed according to standard procedures, by medical personnel at the time of birth. Furthermore, mental health outcomes were assessed twice over an 8-year period, using validated screening instruments. Nevertheless, the findings should be viewed in light of the following limitations. First, placental weight included membranes and the umbilical cord, and since these components are not involved in nutrient exchange, this may in particular affect measurement of the placental-to-birth weight ratio. Second, placental size provides only limited insight into the role of the placenta in fetal programming [8]. Recent work has found that the expression of placental HSD11B2 mRNA is decreased in anxious pregnant women [46]; since increased fetal exposure to cortisol has been associated with neurodevelopmental disorders later in life [30], it is possible that reduced placental HSD11B2 activity provides a link between altered placental function and fetal programming [47]. In animals, reduced HSD11B2 levels have been associated with decreased placental weight [48]; it is of interest to investigate whether this is also the case in humans, which should provide further insight into how placental size relates to function. Third, we only examined ADHD symptoms in adolescence due to limited data availability. Fourth, we did not assess mental health diagnosis, but rather whether children/adolescents screened positive for probable diagnosis based on symptoms. This may help explain why the frequencies of mental health problems in this study appear somewhat high, in particular at 8 years, but are typical at the symptom level for children of this age.

In conclusion, this study shows that placental size was associated with mental health problems in boys during childhood and adolescence. Placental enlargement may occur in response to chronic adverse intrauterine conditions, and could lead to altered fetal brain development, with long-term effects on mental health. Future work is required to determine whether deviation in placental size is causal or lies on the causal pathway linking prenatal exposures to child psychopathology.
